# Early Enriched Environment Exposure Protects Spatial Memory and Accelerates Amyloid Plaque Formation in APP^Swe^/PS1^L166P^ Mice

**DOI:** 10.1371/journal.pone.0069381

**Published:** 2013-07-24

**Authors:** Francesca Montarolo, Roberta Parolisi, Eriola Hoxha, Enrica Boda, Filippo Tempia

**Affiliations:** 1 Institute Cavalieri Ottolenghi (NICO), University of Torino, Orbassano (Torino), Italy; 2 Department of Neuroscience and National Institute of Neuroscience-Italy (INN), University of Torino, Torino, Italy; University of Pittsburgh, United States of America

## Abstract

Enriched environment exposure improves several aspects of cognitive performance in Alzheimer’s disease patients and in animal models and, although the role of amyloid plaques is questionable, several studies also assessed their response to enriched environment, with contrasting results. Here we report that rearing APP^Swe^/PS1^L166P^ mice in an enriched environment since birth rescued the spatial memory impairment otherwise present at 6 months of age. At the same time, the exposure to the enriched environment caused a transient acceleration of plaque formation, while there was no effect on intracellular staining with the 6E10 antibody, which recognizes β-amyloid, full length amyloid precursor protein and its C-terminal fragments. The anticipation of plaque formation required exposure during early development, suggesting an action within critical periods for circuits formation. On the other hand, chronic neuronal activity suppression by tetrodotoxin decreased the number of plaques without affecting intracellular amyloid. These results indicate that enriched environment exposure since early life has a protective effect on cognitive deterioration although transiently accelerates amyloid deposition. In addition, the effects of the enriched environment might be due to increased neuronal activity, because plaques were reduced by suppression of electrical signaling by tetrodotoxin.

## Introduction

Alzheimer’s disease (AD), a severe neurodegenerative disorder, is the most common form of dementia. Affected individuals show progressive memory loss, difficulties in learning, diminished recall accuracy and impaired problem solving [1]. The hallmarks of the disease are amyloid deposits of aggregated β-amyloid (Aβ) peptides and intracellular neurofibrillary tangles composed of hyperphosphorylated tau protein. Aβ is a 38–42 amino acid peptide produced by the sequential cleavage of the amyloid precursor protein (APP) by β- and γ-secretases and its accumulation in brain seems to be the primary early event driving the pathogenesis [2], [3]. Rare familial cases of AD (FAD) are caused by mutations of the amyloid precursor protein gene (*APP)* or of some of the proteins forming the γ-secretase enzyme complex (*PSEN1*, coding for presenilin 1, *PSEN2*, coding for presenilin 2) while the vast majority of AD cases are sporadic, late-onset forms of the disease, suggesting that environmental factors may play a role in etiology. Additionally, a number of epidemiological studies have identified education and advanced occupational attainment as environmental factors that can affect the risk of developing AD. Indeed a reduced risk of developing AD is reported in individuals that follow a lifestyle including enriched social, physical and/or cognitive activities [4]–[8].

In order to study the mechanisms responsible for this protective effect, a recent and growing number of studies has been aimed at assessing the consequences of physical and cognitive activity in mouse models of AD. Rearing animals in an enriched environment (EE) is a noninvasive strategy to enhance neuronal activity *in vivo*, which allowed to establish that brain structure and function are dynamically responsive to experience [9]. In EE, experimental animal models are exposed to a complex array of stimuli (e.g. toys, obstacles and tunnels), and allowed to freely experience voluntary physical activity. This is often further enriched by exposing the animals to an enhanced social environment. Numerous studies indicate that EE promotes plastic modifications of the neuronal circuits, including dendritic growth, axonal branching and spine formation ([10]–[12], for a thorough review of the effects of enriched environment, see [13]). These modifications are more robust if EE is applied in specific time windows during postnatal life, named critical periods, during which neural circuits display a heightened sensitivity to acquire instructive and adaptive signals from the external environment, but some EE-induced plastic phenomena can be detected also during the adulthood.

Previous experiments in adult AD transgenic mice have clearly shown that spatial learning and other cognitive functions are improved by exposure to EE [14]–[19], but they yielded contrasting results regarding the effects of EE on amyloid plaques. Briefly, on one hand, Jankowsky et al. [16], [20] have shown that exposure to EE causes an increase in plaque formation in hippocampus of APP^Swe^/PS1**^Δ^** ^E9^ transgenic mice; on the other hand Lazarov et al. [15] reported a pronounced reduction in cerebral Aβ levels in the same APP^Swe^/PS1**^Δ^** ^E9^ mice, while Arendash et al. [14] observed no change in Aβ deposition in aged APP^Swe^ transgenic mice.

It is likely that the effects of EE are due, at least in part, to experience-dependent increases in neuronal activity, but this issue has not been directly investigated. Different lines of evidence support the idea that neuronal activity may modulate Aβ production and deposition. Neuronal stimulation *in vitro*, positively regulates APP processing at the β–secretase site, inducing an increase in APP-cleavage products with a consequent increase in Aβ production and secretion into the interstitial fluid [21]–[23]. This is in line with the observation that the areas of the human brain with the highest default activity, including the medial temporal lobe, are among the most vulnerable to early AD pathology [24]. While synaptic activity has been shown to affect the rate of Aβ secretion from neurons, decreased levels of intracellular Aβ deposits (possibly due to its release in the extracellular milieu) are associated with protection from the loss of synaptic contacts [25], [26]. The intracellular accumulation of APP/Aβ has been proposed as the early toxic event initiating the cognitive decline [27], [28]. However, the nature of intracellular staining with Aβ antibodies has been questioned, since these studies utilized antibodies that also detect full-length APP or intermediate APP cleavage products, while Aβ-specific antibodies do not result in intracellular labeling [29]. For this reason, it is more correct to refer to APP/Aβ intracellular staining instead of simply intracellular Aβ [29]. Thus, such APP/Aβ intracellular immunoreactivity has been linked to the first cognitive deficits [27], [28]. However, the effect of a physiological neuronal stimulation, as obtained by exposure to an EE, on intracellular APP/Aβ has not been investigated.

Here, we examined the effects of the enhanced neuronal activity induced by EE on amyloid deposition and cognitive performance in APP^Swe^/PS1^L166P^ mice, that have high Aβ_1–42_/Aβ_1–40_ ratio causing an earlier and stronger brain accumulation of amyloid deposits compared to most other murine AD models [30]. In agreement with previous studies we show that, also in APP^Swe^/PS1^L166P^ mice, EE improves spatial memory. Amyloid plaques are transiently increased, but only in mice exposed to EE in a time window that includes the critical periods of experience-dependent circuits formation and refinement. We confirm that these effects may be attributed to neuronal activity, as its suppression causes a significant decrease in plaque deposition. Finally, we show that intracellular APP/Aβ levels are not influenced by neuronal activity, suggesting a minor role of intracellular amyloid deposits in cognitive deterioration.

## Materials and Methods

### 1 Animals

Female hemizygous APP^Swe^/PS1^L166P^ transgenic mice (n = 103) and wild-type littermates (n = 83) were maintained in C57BL/6J genetic background and used for all experimental paradigms. APP^Swe^/PS1^L166P^ double transgenic mice express mutated *APP* (KM670/671NL, Swedish mutation) and *PSEN1* (L166P) under the control of a neuron-specific *Thy1* promoter element [30]. They were obtained from Dr. Mathias Jucker, Hertie-Institute for Clinical Brain Research, University of Tübingen (Germany). The animal experimental procedures were approved by the Bioethical Committee of the University of Torino (July 7, 2010 and September 14, 2011) and by the Italian Ministry of Health (Experimental Protocol Communications of December 6, 2010 and October 17, 2011).

### 2 Histological Procedures

APP^Swe^/PS1^L166P^ and wild-type mice were deeply anesthetized and transcardially perfused with 4% paraformaldehyde in 0.12 M phosphate buffer, pH 7.2–7.4. The brains were removed and immersed in the same fixative at 4°C for 24 hours and then cryoprotected in 30% sucrose in 0.12 M phosphate buffer. Brains were frozen and serially cut by a cryostat in 30 µm-thick coronal sections collected in phosphate buffered saline (PBS). In order to detect amyloid deposits, one series was incubated overnight at 4°C with the monoclonal anti-human β-amyloid antibody (1–16, 6E10, 1∶300; Covance) diluted in PBS with 0.25% Triton X-100 and 1.5% normal goat serum. Another series was incubated with the monoclonal anti-NeuN antibody (MAV377, 1∶1000; Millipore), in order to selectively stain neurons. Immunohistochemical reactions were performed by the avidin–biotin–peroxidase method (Vectastain ABC Elite kit; Vector Laboratories, Burlingame, CA, USA) and revealed using 3,3′-diaminobenzidine (3% in Tris–HCl) as chromogen. Morphometric analysis of the sections was performed by means of the Neurolucida system (MicroBrightField, Williston, VT, USA) and ImageJ software (http://rsbweb.nih.gov/ij/index.html). For extracellular deposition, APP/Aβ positive plaque number (plaque number/mm^2^) and size (µm^2^) were quantified while for intracellular staining we quantified APP/Aβ positive cell number (cell number/mm^2^), size (µm^2^), pixel intensity (optical density, AU) and hippocampal pyramidal cell layer thickness (µm). For each parameter at least 3 sections for animal were evaluated.

### 3 Behavioural Tests

Spatial learning and memory of 2-, 4- and 6-month-old APP^Swe^/PS1^L166P^ and wild-type mice was investigated by means of Morris water maze [31] and eight arm radial maze tests (see Supporting Information Text S1 for details). The mazes were located in a room with numerous extra-maze cues visible to the mice during testing. The behavioral tests were performed by an individual blinded to the animal’s genotype.

### 4 Enriched/Standard Environment Conditions

The enriched environment (EE) consisted of a large cage (80 × 50 × 80 cm) with multiple floors and with a wire mesh lid containing several food hoppers, running wheels and differently shaped and colored objects (i.e., tunnels, shelters, stairs and boxes) that were completely substituted with others once every ten days. The cage housed at least 15–20 mice. The standard environment (SE) consisted of standard laboratory cages (26 × 42 × 18 cm) without objects, housing 4–5 mice. Pregnant hemizygous female APP^Swe^/PS1^L166P^ mice were housed in an EE or SE cage and their offspring remained there until 2, 4 or 6 months of age. At the end of the EE/SE period, APP^Swe^/PS1^L166P^ and wild-type mice underwent behavioral tests and then histological procedures. In addition, a group of APP^Swe^/PS1^L166P^ mice housed in SE for the first two months of life was exposed to EE until four months of age and then analyzed. The control groups were represented by APP^Swe^/PS1^L166P^ and wild-type mice reared in SE since birth until four months of age.

### 5 Micro-osmotic Pump Implantation

APP^Swe^/PS1^L166P^ mice of 2 months of age were anaesthetized with an intraperitoneal injection of ketamine (100 mg/kg; Ketavet; Bayer, Leverkusen, Germany) supplemented by xylazine (5 mg/kg; Rompun; Bayer, Leverkusen, Germany) and placed in a stereotaxic apparatus. After stereotaxic determination a small hole was obtained using a high-speed dental drill. A 28 gauge stainless steel cannula was inserted into the dorsal hippocampus (with the tip reaching 1.5 mm below the surface) and fixed to the skull by dental cement. The cannula was connected by a polyvinyl chloride tubing to a micro-osmotic pump (Alzet model 1004, Cupertino, CA, USA), containing 10 mM tetrodotoxin (TTX, Tocris Bioscience, United Kingdom) dissolved in a saline solution (n = 6). All control animals received the saline solution alone (vehicle, n = 6). The stereotaxic coordinates for the cannula implantation were determined according to the atlas of Paxinos & Franklin [32]: 1.82 mm posterior to the bregma and 1 mm lateral to the midline. According to the manufacturer’s standards, these micro-osmotic pumps infuse solutions at 0.11 µl/hr for 28 days. After securing the cannula, the connected micro-osmotic pump was inserted into a preformed subcutaneous pocket along the back. Finally, mice were returned to their cages with water and food *ad libitum*. At the end of the experiments, the animals were sacrificed to proceed with the histological analysis.

### 6. Statistics

Data are presented as mean value ± SEM. Unless otherwise indicated, n = number of animals. Statistical significance was assessed by Student’s *t*-test, One-way ANOVA and Two-ways Repeated-Measures ANOVA, as appropriate. All the graphs were designed using Graph Pad Prism software (San Diego California, USA) and statistical tests were performed by means of SPSS software (SPSS Inc., Chicago, IL, USA). *P* values lesser than 0.05 were accepted as significant.

## Results

### 1. Age-related Amyloid Deposition in APP^Swe^/PS1^L166P^ Mice

**Figure 1:**
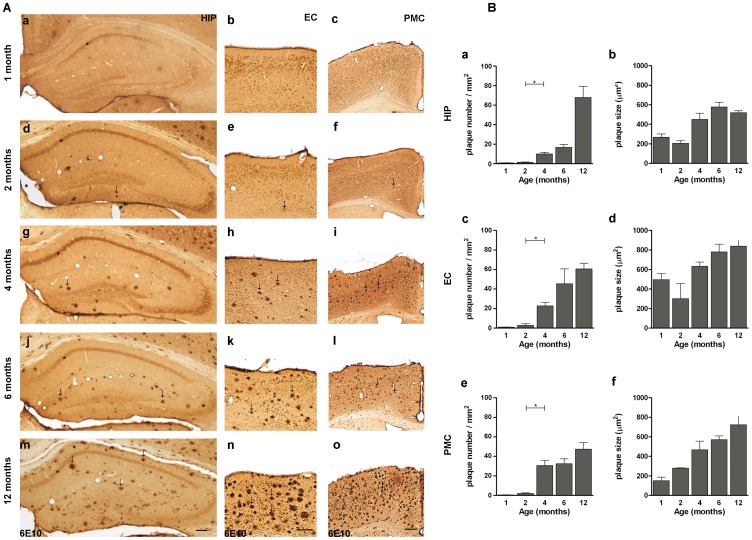
Age-related amyloid deposition in APP^Swe^/PS1^L166P^ mice. **(A)** Representative images of coronal sections of hippocampus (**a**; **d**; **g**; **j**; **m**), entorhinal (**b**; **e**; **h**; **k**; **n**) and primary motor (**c**; **f**; **i**; **l**; **o**) cortex of 1- (**a**; **b**; **c**), 2- (**d**; **e**; **f**), 4- (**g**; **h**; **i**), 6- (**j**; **k**; **l**) and 12- (**m**; **n**; **o**) month-old APP^Swe^/PS1^L166P^ mice, immunostained with 6E10 antibody. Note APP/Aβ intracellular immunoreactivity of the pyramidal cell layer in the CA1 region of hippocampus (*), already present at 1 month of age, and the presence of small extracellular amyloid deposits (some examples are indicated by arrows) at 2 months. Note also the presence of compact extracellular amyloid deposits (arrows) beginning from 4 months. At 12 months of age, these amyloid deposits cover a large area of hippocampus, entorhinal and primary motor cortex while the APP/Aβ intracellular staining is weak. Calibration bar, 300 µm. **(B)** Quantitative analysis of extracellular amyloid load in hippocampus (**a**; **b**), entorhinal (**c**; **d**) and primary motor cortex (**e**; **f**) in terms of plaque number (**a**; **c**; **e**) and plaque size (**b**; **d**; **e**) in 1-, 2-, 4-, 6- and 12-month-old APP^Swe^/PS1^L166P^ mice. The analysis showed an increase beginning from 2 to 12 months of age for hippocampus (**a**; **b**), entorhinal cortex (**c**; **d**) and primary motor cortex (**e**; **f**) in terms of plaque number (**a**; **c**; **e**) and plaque size (**b**; **d**; **f**) (n = 5 in each group, three sections per mouse, three frames per section, * P<0.05, One-way ANOVA). Error bars represent SEM. (HIP, hippocampus; EC, entorhinal cortex; PMC, primary motor cortex).

**Figure 2:**
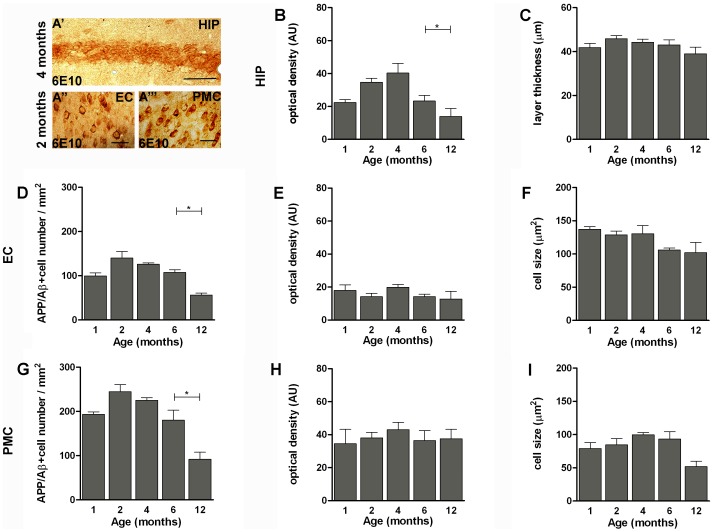
Time course of intracellular APP/Aβ immunoreactivity in APP^Swe^/PS1^L166P^ mice. Representative images of coronal sections of hippocampal pyramidal cell layer (**A**′), entorhinal (**A′′**) and primary motor (**A′′′**) cortex of APP^Swe^/PS1^L166P^ mice, at the peak of intracellular 6E10 immunostaining positivity. Calibration bar, 50 µm. (**B**-**I**) Quantitative analysis of APP/Aβ immunoreactivity in the intracellular compartment in hippocampal pyramidal cell layer (**B**; **C**), entorhinal (**D**; **E**; **F**) and primary motor cortex (**G**; **H**; **I**) in terms of APP/Aβ+ optical density (**B**; **E**; **H**), hippocampal pyramidal cell layer thickness (**C**), cell number (**D**; **G**) and cell size (**F**; **I**) in 1-, 2-, 4-, 6- and 12-month-old APP^Swe^/PS1^L166P^ mice. The APP/Aβ+ optical density of the intracellular compartment in hippocampal pyramidal cell layer (**B**) and the APP/Aβ+ cell number in both entorhinal (**D**) and primary motor (**G**) cortex showed age-related differences. The hippocampal pyramidal cell layer thickness (**C**) and the APP/Aβ+ cell size in both entorhinal (**F**) and primary motor cortex (**I**) showed no age-related differences (n = 5 in each group, three sections per mouse, three frames per section, * *P*<0.05, One-way ANOVA). Error bars represent SEM. (AU, arbitrary units; HIP, hippocampus; EC, entorhinal cortex; PMC, primary motor cortex).

**Figure 3:**
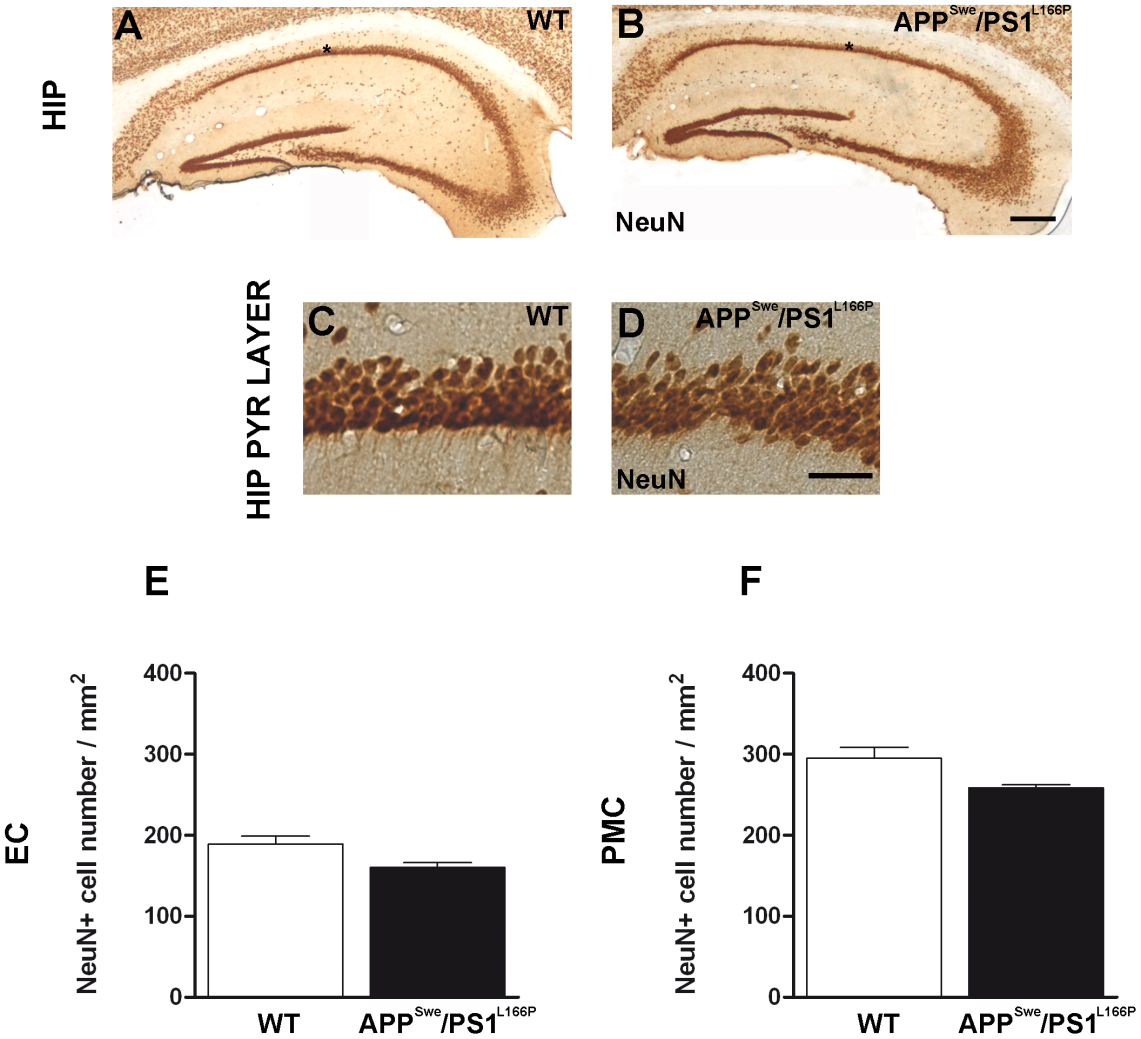
Twelve-month-old APP^Swe^/PS1^L166P^ mice do not reveal a reduction in neuronal cell number. Representative images of coronal hippocampal sections of 12 month old wild-type (**A**; **C**) and APP^Swe^/PS1^L166P^ (**B**; **D**) mice stained with the neuron-selective NeuN antibody. The images show that there is no evident cell loss at 12 months of age in the hippocampus of APP^Swe^/PS1^L166P^ mice. Calibration bars, 250 µm (**A**; **B**); 25 µm (**C**; **D**). The analysis of NeuN immunostaining in entorhinal (**E**) and primary motor (**F**) cortex of wild-type and APP^Swe^/PS1^L166P^ mice revealed that also in these structures there is no significant loss of cells at 12 months of age (n = 5 in each group, three sections per mouse, three pictures per sections, Student’s *t*-test). Error bars represent SEM. (HIP, hippocampus; HIP PYR LAYER; hippocampal pyramidal layer; EC, entorhinal cortex; PMC, primary motor cortex).

**Figure 4:**
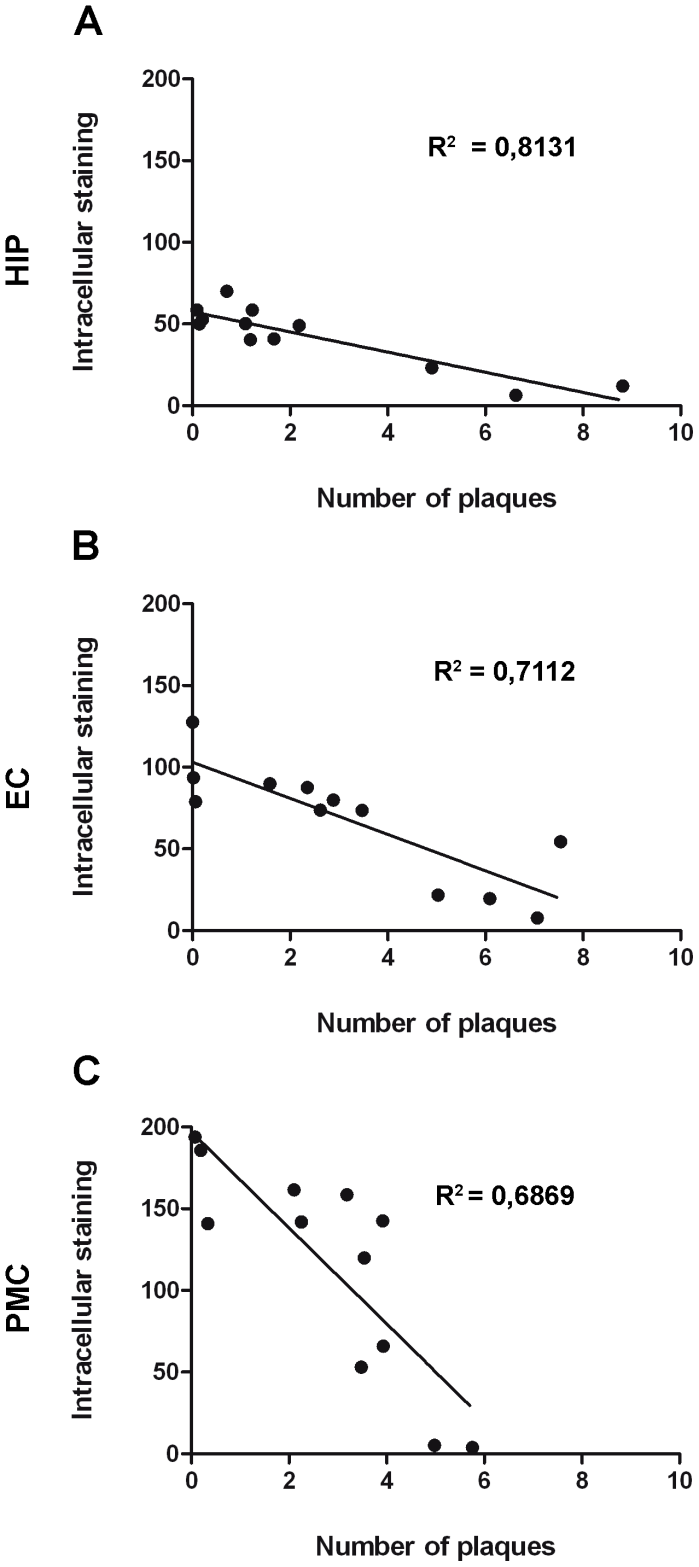
Inverse correlation between intra- and extracellular APP/Aβ immunoreactivity in APP^Swe^/PS1^L166P^ mice. A significant inverse relationship exists between intracellular APP/Aβ immunoreactivity and number of amyloid plaques in APP^Swe^/PS1^L166P^ mice of different ages in hippocampus (**A**), entorhinal (**B**) and primary motor cortex (**C**). In hippocampus intracellular staining is represented by the optical density of 6E10 staining in the pyramidal cell layer; in entorhinal and primary motor cortex intracellular staining is represented by APP/Aβ+ cell number/mm^2^. (HIP, hippocampus; EC, entorhinal cortex; PMC, primary motor cortex).

Previous work showed that by 2 months of age hemizygous APP^Swe^/PS1^L166P^ mice develop a severe Aβ_1–42_-driven cerebral amyloidosis, with amyloid lesions typical of AD spreading from the cortical areas to the rest of the brain [30]. As a first step, we characterized the time course of amyloid immunoreactivity in APP^Swe^/PS1^L166P^ mice from 1 to 12 months of age. The 6E10 antibody used in this study recognizes an epitope within the Aβ domain (Aβ_1–16_) and therefore it detects both full-length APP and APP cleavage products containing the Aβ domain, including the Aβ_1–40/42_ peptide [29]. We refer to such 6E10 targets as APP/Aβ [29]. The progressive formation of Aβ plaques and intracellular deposits was studied in hippocampus and entorhinal cortex because they are the earliest and most severely affected areas in AD, and functional deficits in these structures are related to the first symptoms of memory impairment in AD patients [33]. We also analyzed the primary motor cortex, which is relatively spared both in early and late onset AD [34], [35] while in APP^Swe^/PS1^L166P^ mice shows a strong expression of the transgenes [30]. A progressive deposition of extracellular Aβ in plaques was detected in APP^Swe^/PS1^L166P^ brain (Fig. 1). Notably, amyloid plaques were almost absent in 1-month-old APP^Swe^/PS1^L166P^ (hippocampus: 0.8±0.1 plaque number/mm^2^; entorhinal cortex: 0.2±0.1 plaque number/mm^2^; primary motor cortex: 0.5±0.1 plaque number/mm^2^) and beginning from 2 months of age (hippocampus: 1.4±0.1 plaque number/mm^2^; entorhinal cortex: 2.8±0.3 plaque number/mm^2^; primary motor cortex: 2.0±0.7 plaque number/mm^2^) they showed a progressive increase until the last time point analysed of 12 months (hippocampus: 66.8±11.3 plaque number/mm^2^; entorhinal cortex: 60.6±5.8 plaque number/mm^2^; primary motor cortex: 47.4.±7.0 plaque number/mm^2^). The largest increase of APP/Aβ positive plaques number and size occurred between 2 and 4 months (Fig. 1). The extracellular deposition was preceded by intracellular APP/Aβ immunoreactivity (Fig. 2A). Although at later time points some APP/Aβ positive astrocytes and microglial cells could be detected (data not shown and ref. [36]), within the time window analyzed in our work APP/Aβ intracellular immunoreactivity exclusively pertained to neuronal somata (Fig. 2A). In the hippocampus, the APP/Aβ intracellular immunoreactivity was confined to the pyramidal cell layer, especially in the CA1 region, showing a compact APP/Aβ staining (Fig. 1, asterisk and Fig. 2A′). In the hippocampal CA1 pyramidal layer, the intensity of the intracellular APP/Aβ staining peaked at 4 months of age (optical density: 40.29±5.77 AU) (Fig. 2B), while the number and size of the APP/Aβ-positive cells, (quantified as the thickness of the 6E10 labelled layer due to the high compactness/cellularity of such area), did not change throughout the mouse lifespan (Fig. 2C). In the entorhinal and primary motor cortices, the intracellular amyloid accumulation developed with a parallel time course, without any significant difference (Two way ANOVA, *P*>0.05*)*. In these areas, in 1-month-old APP^Swe^/PS1^L166P^ mice a significant number of cells showed intracellular APP/Aβ reactivity (entorhinal cortex: 99.60±6.96 cells/mm^2^; primary motor cortex: 193.33±5.85 cells/mm^2^). At 2 months the number of labelled cells was higher (entorhinal cortex: 140.07±14.43 cells/mm^2^; primary motor cortex: 244.51±16.48 cells/mm^2^), but thereafter it began to decline (4 and 6 months) reaching low levels at 12 months (entorhinal cortex: 56.35±4.32 cells/mm^2^; motor cortex: 91.78±16.14 cells/mm^2^; Fig. 2D, G). The cells labelled by the 6E10 antibody showed no changes in size (Fig. 2F; 2I) or intracellular APP/Aβ content (optical density of the staining; Fig. 2E; 2H) either in entorhinal (Fig. 2D–F) or in primary motor cortex (Fig. 2G–I). The reduction of the number of APP/Aβ positive cells was not attributable to cell loss. In fact, 12-month–old APP^Swe^/PS1^L166P^ mice didn’t show any significant decrease in the total number of neurons relative to their wild-type controls (Fig. 3). To better quantify the relationship between the intracellular and the extracellular amyloid pools, the intracellular APP/Aβ immunoreactivity was correlated to the number of amyloid plaques in 2-, 4-, 6- and 12-month-old APP^Swe^/PS1^L166P^ mice. Amyloid deposition in APP^Swe^/PS1^L166P^ mice showed a significant inverse correlation between these two pools in hippocampus (R^2^ = 0.8131, *P*<0.0001; Fig. 4A), entorhinal (R^2^ = 0.7112, *P* = 0.0006; Fig. 4B) and primary motor cortex (R^2^ = 0.6869, *P* = 0.0009; Fig. 4C).

### 2. Spatial Learning and Memory in APP^Swe^/PS1^L166P^ Mice

**Figure 5:**
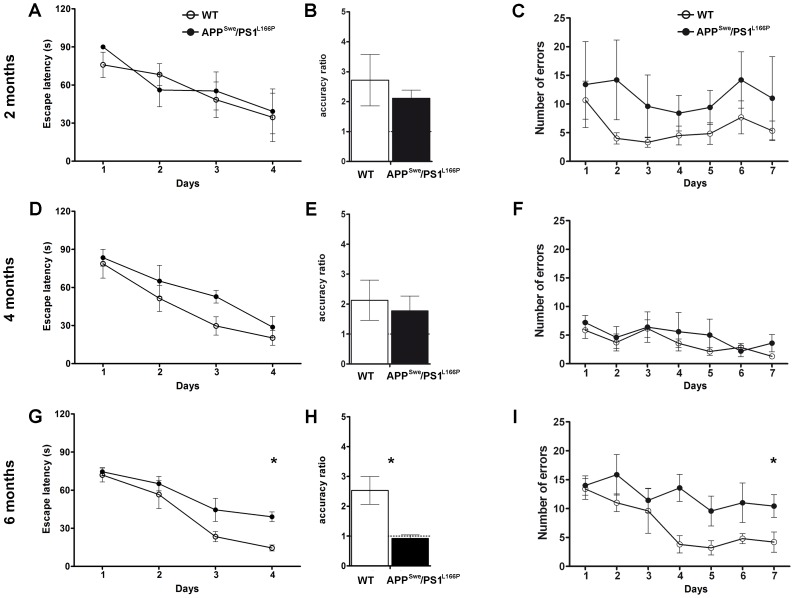
Six-month-old APP^Swe^/PS1^L166P^ mice show a defect in spatial learning and memory compared to their wild-type littermates. Left panels: Morris water maze test. Average spatial memory performance during the acquisition phase expressed as escape latency over four daily sessions of four consecutive trials in 2- (A), 4- (D) and 6- (G) month-old wild-type and APP^Swe^/PS1^L166P^ mice (n = 8 in each group, * P<0.05 genotype effect, Two-way Repeated-Measures ANOVA). Average accuracy ratio of 2- (B), 4- (E) and 6- (H) month-old wild-type and APP^Swe^/PS1^L166P^ mice tested in the probe trial 24 h after the last acquisition session (day 5) (n = 8 in each group, *: P<0.05, Student’s *t*-test). Right panels: eight radial arm maze test. The number of errors is plotted versus the day of testing in 2- (C), 4- (F) and 6- (I) month-old wild-type and APP^Swe^/PS1^L166P^ mice. (n = 8 in each group, * *P*<0.05 genotype effect, Two-way Repeated-Measures ANOVA). Error bars represent SEM. (WT, wild-type).

Spatial memory was tested in 2-, 4- and 6-month-old APP^Swe^/PS1^L166P^ and wild-type mice by means of the Morris water maze and the eight radial arm maze tests (Fig. 5).

#### 2.1. Morris water maze test

The acquisition phase of the Morris water maze test (Fig. 5A, D, G) assessed the ability of the mice to acquire spatial information to learn the location of the hidden platform, which remained at the same place on all trials and days. In the acquisition phase the escape latency of 2- (Fig. 5A) and 4-month-old (Fig. 5D) APP^Swe^/PS1^L166P^ was not different from that of the wild-type mice: for both groups the escape latency significantly decreased over time, indicating that they were able to learn across days (Two-way Repeated-Measures ANOVA, genotype effect, *P*>0.05; day effect, *P*<0.001 for 2 and 4 months of age). Conversely, 6-month-old APP^Swe^/PS1^L166P^ mice behaved differently from the wild-type mice of the same age (Two-way Repeated-Measures ANOVA, genotype effect, *P*<0.05; day effect, *P*<0.001) (Fig. 5G). On the last day of training (day 4), they spent significantly more time to reach the submerged platform relative to wild-type (escape latency: 14.50±1.50 s for wild-type; 39.04±0.93 s for APP^Swe^/PS1^L166P^; Student’s *t*-test and Bonferroni post-hoc test, *P*<0.05), indicating a significant spatial memory defect in 6-month-old APP^Swe^/PS1^L166P^ mice.

The fifth day, the platform was removed and all mice were tested for the ability to remember the location of the hidden platform during a single probe trial, which lasted 60 s (Fig. 5B, E, H). The retention was measured by the accuracy ratio (AR; time spent in the target quadrant multiplied by 3 and divided by the time spent in the others three quadrants; see Supporting Information Text S1 for details). As shown in figure 5H, in the probe trial the mean AR obtained by 6-month-old APP^Swe^/PS1^L166P^ mice was significantly different from that of the wild-type mice (Student’s *t*-test, *P* = 0.006). In fact, 6-month-old APP^Swe^/PS1^L166P^ mice spent the same time in the target quadrant as in each of the other quadrants, so that the AR was not significantly different from the chance level of 1 (AR = 0.92±0.11, Student’s *t-* test, *P*>0.05). In contrast, wild-type mice showed an AR greater than 2, significantly different from the value of their APP^Swe^/PS1^L166P^ littermates (2.53±0.47, Student-*t* test, *P* = 0.0008). These results indicate the presence of a learning impairment in 6-month-old APP^Swe^/PS1^L166P^ mice. On the contrary, the AR obtained by both transgenic and wild-type 2- and 4-month-old mice was comparable (Fig. 5B, E; Student’s *t*-test, *P*>0.05). During the test there was no difference in swimming velocity or distance covered (data not shown) between all genotype groups and at all analyzed time points, suggesting normal vision, locomotor skills, and motivation in APP^Swe^/PS1^L166P^ animals.

#### 2.2. Eight radial arm maze test

In order to confirm the memory deficit found in 6-month-old APP^Swe^/PS1^L166P^ mice, spatial learning and memory were also assessed in the eight radial arm maze by counting the number of errors (entry to previously visited arms; see Supporting Information Text S1 for details) across 7 testing days (Fig. 5C, F, I). The statistical analysis revealed a significant defect in 6-month-old APP^Swe^/PS1^L166P^ mice relative to their wild-type littermates (Two-way Repeated-Measures ANOVA, genotype effect, *P*<0.05; day effect, *P*<0.05; number of errors 10.12±1.73 for APP^Swe^/PS1^L166^, 6.00±2.30 for wild-type; Bonferroni: *P*<0.05) (Fig. 5I). Similar to what observed in the Morris water maze, in this test 2- and 4-month-old APP^Swe^/PS1^L166P^ mice did not show statistically significant differences relative to the wild-types (Two-way Repeated-Measures ANOVA, genotype effect, P>0.05; day effect, P>0.05 for 2 and 4 months of age) (Fig. 5C, F). These results, in agreement with the data obtained in the Morris water maze test, confirm the appearance, between 4 and 6 months of age, of a spatial memory deficit in APP^Swe^/PS1^L166P^ transgenic mice, not present in wild-type control animals.

### 3. Effects of the Enriched Environment on Spatial Memory

Animals living in an enriched environment (EE) are exposed to higher levels of sensory and social stimuli, and exhibit enhanced motor activity and inter-individual interaction. These factors are associated with a more intense activity of most brain regions, including those involved in Alzheimer’s disease. Pregnant female APP^Swe^/PS1^L166P^ and wild-type mice were divided into two groups, either reared in standard (SE) or enriched (EE) environmental conditions. Newborn female APP^Swe^/PS1^L166P^ and wild-type mice were maintained in SE or EE until 2, 4 or 6 months, when they underwent the Morris water maze test and then were sacrificed for immunohistochemical analysis.

**Figure 6:**
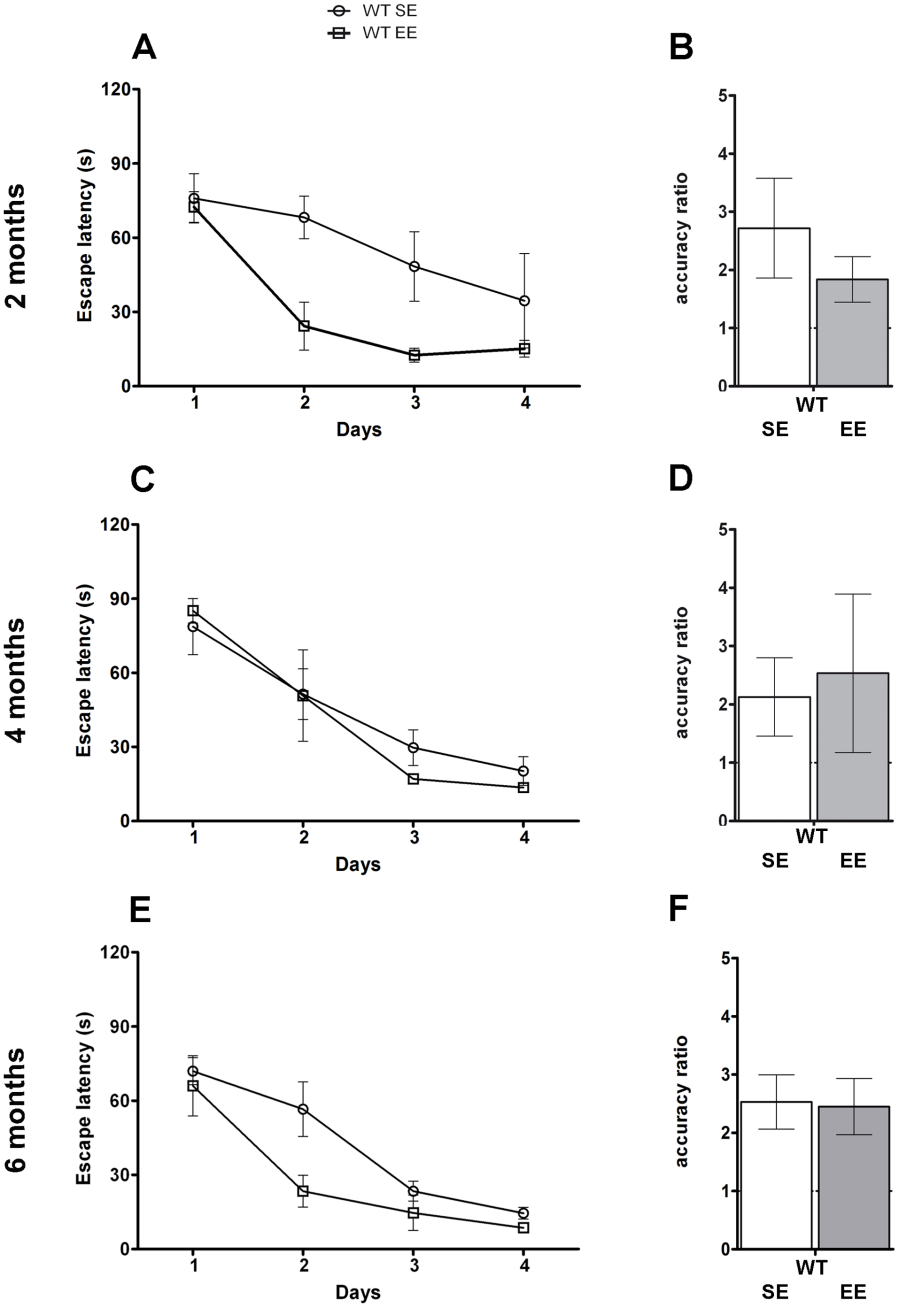
EE does not influence the final performance of wild-type mice in the Morris water maze. Average spatial memory performance during the acquisition phase expressed as escape latency over four daily sessions of four consecutive trials in 2- (**A**), 4- (**C**) and 6- (**E**) month-old wild-type mice housed in SE or EE (n = 8 in each group, Two-way Repeated-Measures ANOVA). Average accuracy ratio of 2- (**B**), 4- (**D**) and 6- (**F**) month-old wild-type mice housed in SE and in EE mice tested in the probe trial 24 h after the last acquisition session (day 5) (n = 8 in each group, Student’s *t*-test). Error bars represent SEM. (WT SE, wild-type mice housed in Standard Environment; WT EE, wild-type mice housed in Enriched Environment).

The analysis of the Morris water maze test revealed that both wild-type mice reared in SE and EE were able to improve their performance across days (Fig. 6; Two-way Repeated-Measures ANOVA, housing condition effect, *P*>0.05; day effect, *P*<0.001). However, 2- and 6-month-old wild-type mice reared in EE showed an improved learning at day 2, as they spent significantly less time to reach the submerged platform relative to those caged in SE (escape latency for 2-month-old wild-type mice 68.18±8.57 s in SE, 24.31±9.72 s in EE; for 6-month-old wild-type mice 56.57±9.76 s in SE, 23.42±8.04 s in EE; Bonferroni, *P*<0.05). In the probe trial, the mean AR did not show any significant difference in wild-type reared in EE relative to SE (Student’s *t-*test, *P*>0.05; Fig. 6B, D, F).

**Figure 7:**
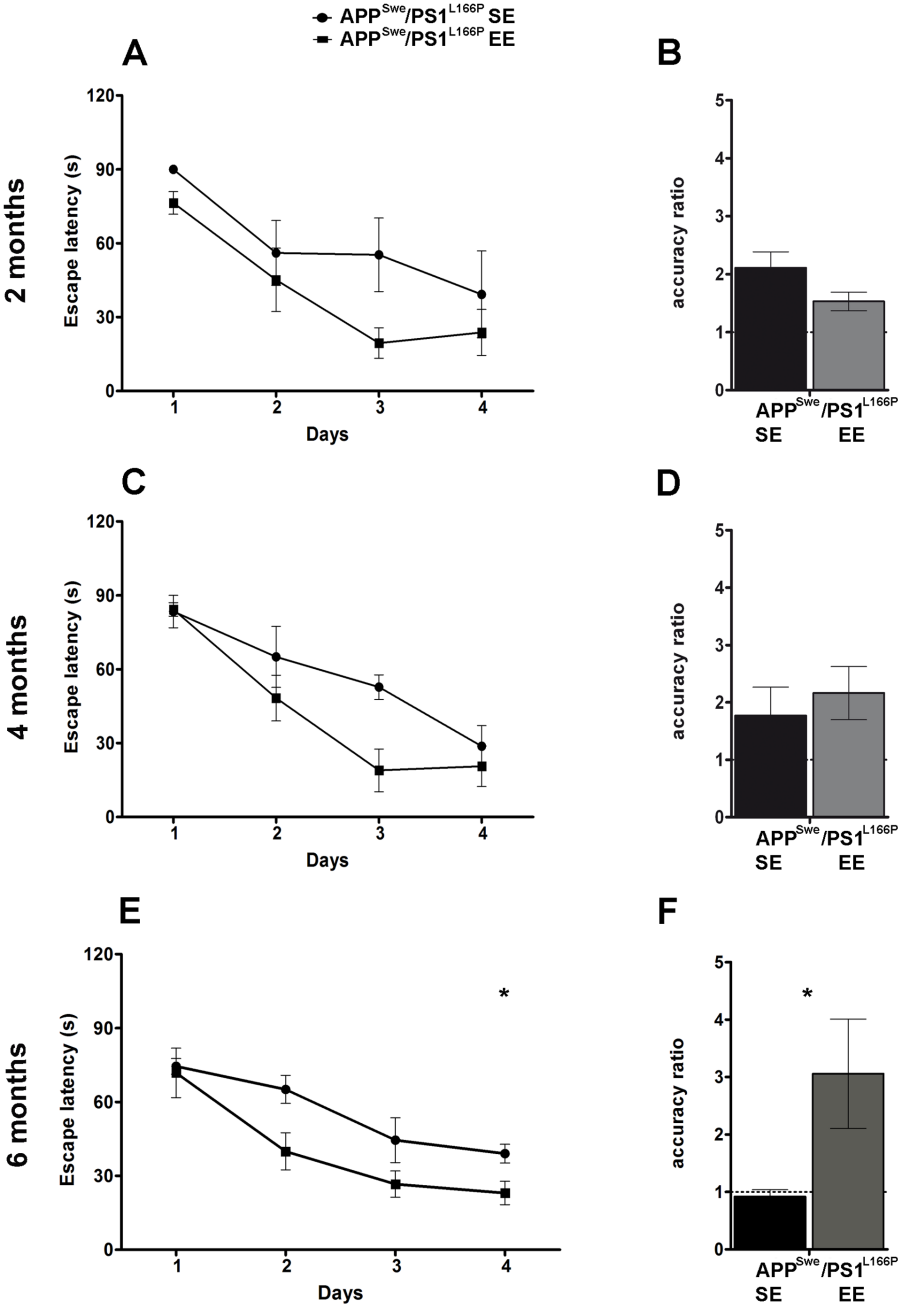
EE reduces the performance deficits of APP^Swe^/PS1^L166P^ mice in the Morris water maze. Average spatial memory performance during the acquisition phase expressed as escape latency over four daily sessions of four consecutive trials in 2- (**A**), 4- (**C**) and 6- (**E**) month-old APP^Swe^/PS1^L166P^ mice housed in SE or EE (n = 8 in each group, * P<0.05 housing condition effect, Two-way Repeated-Measures ANOVA). Average accuracy ratio of 2- (**B**), 4- (**D**) and 6- (**F**) month-old APP^Swe^/PS1^L166P^ mice housed in SE and in EE, tested in the probe trial 24 h after the last acquisition session (day 5) (n = 8 in each group, * P<0.05, Student’s *t*-test). Error bars represent SEM. (APP^Swe^/PS1^L166P^ SE, APP^Swe^/PS1^L166P^ mice housed in Standard Environment; APP^Swe^/PS1^L166P^ EE, APP^Swe^/PS1^L166P^ mice housed in Enriched Environment).

Most notably EE completely reverted the spatial memory deficit displayed by 6-month-old APP^Swe^/PS1^L166P^ mice reared in SE (Fig. 7). In fact, after 6 months spent in EE, APP^Swe^/PS1^L166P^ mice significantly improved their performance (Two-way Repeated-Measures ANOVA, housing condition effect, *P*<0.05; day effect, *P*<0.001). The *post-hoc* test revealed that, since the second day of training, APP^Swe^/PS1^L166P^ mice reared in EE spent significantly less time to reach the submerged platform relative to those which lived in SE (escape latency for APP^Swe^/PS1^L166P^ mice 65.08±4.54 s in SE, 39.95±7.51 s in EE; Bonferroni, *P*<0.05). The memory deficit reversal in 6-month-old APP^Swe^/PS1^L166P^ mice reared in EE was confirmed by a significant improvement in AR relative to SE mice, with an increase from the chance level of about 1 to a value greater than 3 (Fig. 7; Student’s *t-*test, *P*<0.05). On the contrary, in 2- and 4-month-old APP^Swe^/PS1^L166P^ mice, the AR was higher than chance level in both SE and EE groups, without any significant difference between the two housing conditions (Fig. 7B, D; Student’s *t*-test, *P*>0.05). The effect of EE on the escape latency cannot be attributed to other factors like swimming velocity or distance covered, because these parameters showed significant differences neither between genotypes nor between caging conditions at any analyzed time point, suggesting equivalent vision, locomotor skills, and motivation (data not shown).

### 4. Effects of the Enriched Environment on Amyloid Deposition in APP^Swe^/PS1^L166P^ Mice

**Figure 8:**
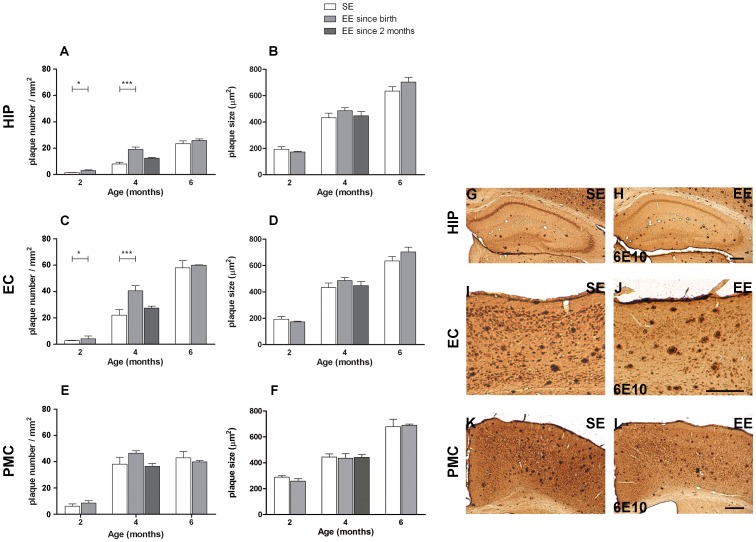
EE causes an anticipation of the extracellular amyloid deposition in hippocampus and entorhinal cortex of APP^Swe^/PS1^L166P^ mice. Left panels: quantitative analysis of amyloid load in the extracellular compartment in hippocampus (A; B), entorhinal (C; D) and primary motor cortex (E; F) in terms of plaque number (A; C; E) and plaque size (B; D; E) in 2-, 4- and 6-month-old APP^Swe^/PS1^L166P^ mice after SE or EE. The analysis showed a significantly higher number of plaques after 2 and 4 months spent in EE since birth for hippocampus (A) and entorhinal cortex (C) but not for primary motor cortex (E). No significant difference was present in terms of plaque size in hippocampus (B), entorhinal (D) or primary motor cortex (F) (n = 5 in each group, three sections per mouse, three frames per section, * P<0.05, *** *P*<0.001, Student’s *t*-test). Error bars represent SEM. Right panels: representative images of coronal hippocampal (G; H), entorhinal (I; J) and primary motor cortical (K; L) sections of 4-month-old APP^Swe^/PS1^L166P^ mice reared in SE (G; I; K) or in EE (H; J; L), immunostained with 6E10 antibody. Note the higher number of plaques in hippocampus and in entorhinal cortex after 2 or 4 months spent in EE. The same difference is not present in primary motor cortex. Calibration bars, 300 µm. (HIP, hippocampus; EC, entorhinal cortex; PMC, primary motor cortex; SE, Standard Environment; EE, Enriched Environment).

We next asked whether exposure to the EE affected the deposition of amyloid in mouse brain (Fig. 8 and Table S1 Supporting Information). The hippocampus, the entorhinal and the primary motor cortex of same mice tested for spatial memory in the Morris water maze test were analyzed. At 2 and 4 months, mice reared in the EE showed, in hippocampus and entorhinal cortex, a higher number of amyloid positive plaques relative to controls grown in standard cages (Fig. 8A; 8C). At 2 months the number of plaques increased from 1.4±0.1 plaques/mm^2^ to 3.1±0.5 plaques/mm^2^ in hippocampus (Student’s *t*-test, *P*<0.05) and from 2.8±0.3 plaques/mm^2^ to 4.2±2.1 plaques/mm^2^ in the entorhinal cortex (Student’s *t*-test, *P*<0.05). Also at 4 months the difference was present with an increase from 8.1±1.2 plaques/mm^2^ to 19.1±1.8 plaques/mm^2^ in hippocampus (Student’s *t*-test, *P*<0.001) and from 22.1±4.2 plaques/mm^2^ to 40.6±3.8 plaques/mm^2^ in the entorhinal cortex (Student’s *t*-test, *P*<0.001). In contrast, in the primary motor cortex the number of plaques was not significantly affected by the environment at any age (Student’s *t*-test, *P*>0.05 in all ages and conditions), although there was a mild tendency to increase at 4 months (Fig. 8E). Surprisingly, at 6 months these differences were no more present, and mice reared in EE had the same plaque number as those grown in SE (Student’s *t*-test, *P*>0.05 in all regions) (Fig. 8A, C, E). These data indicate that the exposure to the EE caused an anticipation of the deposition of amyloid plaques but this effect was over at 6 months of age. The dimension of amyloid positive plaques was not affected by the environment at any age (Student’s *t*-test, *P*>0.05 in all regions) (Fig. 8B, D, F).

In order to test the role of age in EE capacity to modulate the rate of plaque formation, a separate group of animals was reared since birth until 2 months in SE, and then moved for other 2 months in the EE. These mice showed no difference in plaque number relative to controls continuously reared in SE (Fig. 8A–F). This suggests that the anticipation of plaques formation requires exposure to the EE during development, within the critical periods for nervous system plasticity.

Opposite to the changes observed for the number of extracellular amyloid positive plaques, the intracellular APP/Aβ immunoreactivity was not affected by the EE at any age. Neither the number of positive cells nor the optical density of intracellular labeling nor the thickness of labeling in the hippocampal pyramidal cell layer nor the size of the stained cells were significantly different in the EE and SE groups of animals (Student’s *t*-test, *P*>0.05 for all parameters, regions and ages, see Table S1 Supporting Information).

**Figure 9:**
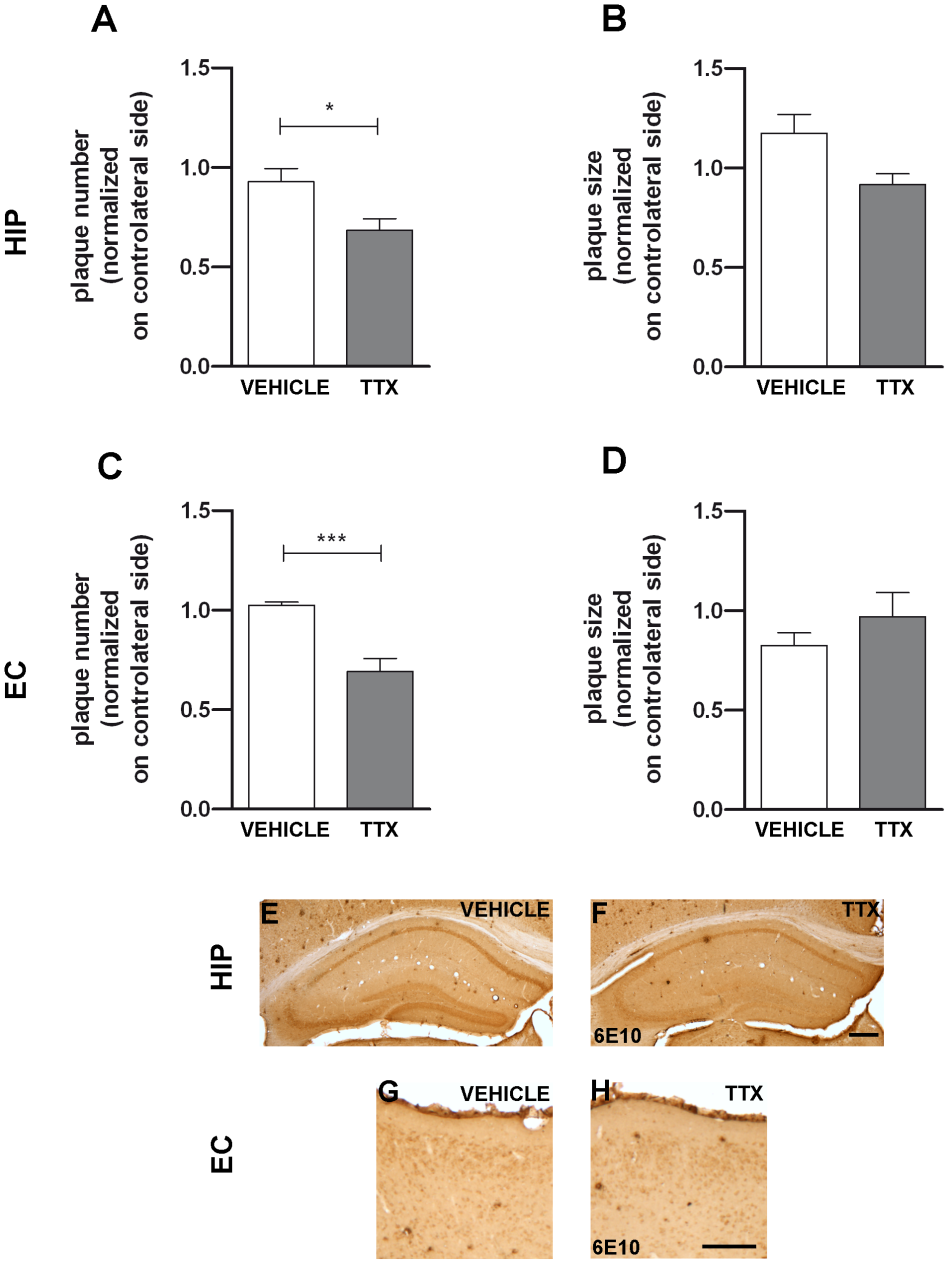
Chronic neuronal activity inhibition by TTX reduces extracellular amyloid in APP^Swe^/PS1^L166P^ mice. Upper panels: quantitative analysis of amyloid load in the extracellular compartment in hippocampus (**A**; **B**) and entorhinal cortex (**C**; **D**) in terms of plaque number (**A**; **C**) and plaque size (**B**; **D**) in APP^Swe^/PS1^L166P^ mice treated with TTX or vehicle alone. The analysis showed an increase of plaque number (**A**; **C**) but not of plaque size (**B**; **D**) after TTX administration in hippocampus (**A**; **B**) and entorhinal cortex (**C**; **D**) (n = 5 in each group, three sections per mouse, three frames per section, * P<0.05, *** *P*<0.001, Student’s *t*-test). Error bars represent SEM. Lower panels: representative images of coronal hippocampal (**E**; **F**) and entorhinal cortical (**G**; **H**) sections of APP^Swe^/PS1^L166P^ mice treated with vehicle (**E**; **G**) or TTX (**F**; **H**), immunostained with 6E10 antibody. Note the decrease in the number of plaques in hippocampus and in entorhinal cortex after TTX. Calibration bars, 300 µm. (HIP, hippocampus; EC, entorhinal cortex).

In order to test whether the effect of the EE on plaque number may be related to increased levels of neuronal activity, in a separate group of mice the electrical signaling via action potentials was abolished by chronic infusion, in the dorsal hippocampus, of tetrodotoxin (TTX), a voltage-dependent sodium channel blocker. Cytochrome oxidase histochemical reaction revealed that neuronal activity was inhibited in the dorsal hippocampus and associated cortices, including the entorhinal cortex (data not shown). After 28 days of continuous TTX infusion, APP/Aβ labeling was analyzed and compared to that found in mice infused with the vehicle. TTX-treated animals showed a significantly lower number of plaques both in hippocampus (Fig. 9A, E, F; Student’s *t*-test, *P*<0.05) and in the entorhinal cortex, with a reduction of 27% and 32% respectively (Fig. 9C, G, H; Student’s *t*-test, *P*<0.001). Similarly to what found after EE, the plaque size (Fig. 9B, D; Student’s *t*-test, *P*>0.05) and the intracellular labeling (Student’s *t*-test, *P*>0.05 for all regions and conditions, see Table S2 Supporting Information) were not affected by TTX infusion.

## Discussion

The present study focused on the effects of early exposure to EE in APPSwe/PS1L166P mice. This experimental paradigm on one hand improved cognitive functions, tested by spatial memory performance; on the other hand it caused a transient acceleration of the formation of extracellular Aβ deposits. These results indicate that environmental factors can modulate the sympomatological and pathological progression in the APP^Swe^/PS1^L166P^ mouse model. A large body of evidence indicates that enhanced physical and/or cognitive activity improves learning and cognitive functions in AD model mice [14]–[19], [37], [38]. We find that the onset of the spatial memory impairment in standard-housed female APP^Swe^/PS1^L166P^ mice is at 6 months of age, several months after the first appearance of amyloid plaques. This result is in agreement with the first report about these animals, in which memory deficits were found at 8 but not at 5 months of age [30]. We found that transgenic mice born and always reared in an EE are protected from APP^Swe^/PS1^L166P^ memory impairment, although at 6 months of age all parameters regarding amyloid deposits are indistinguishable from those found in control animals reared in SE. This result confirms the known dissociation between amyloid burden and cognitive deterioration, which has been noticed also in patients [39]. For this reason it has been proposed that amyloid plaques might be protective, since they sequester toxic forms of Aβ aggregates, most notably soluble oligomers [40].

Interestingly, the protection from cognitive impairment, conferred by EE takes place following a prolonged period in which the number of plaques was even higher than in control animals. This transient elevation in the number of plaques was significant in structures implied in cognitive memory, like hippocampus and entorhinal cortex. At present it is not clear whether there is a relation between such accelerated amyloid deposition in plaques and the subsequent cognitive performance. These data are consistent with the concept that brain areas with the highest default activity, like hippocampus and entorhinal cortex, are among the most affected regions in AD [24].

Previous experiments found very different effects of EE on amyloid deposition, plaque burden and Aβ levels. A decrease of Aβ and plaques was described after physical activity [41], [42], cognitive stimulation [17], [19], [43], [44] or when both aspects of enrichment coexist [15]. However, other studies found either no change [14], [43], [45], [46] or even an increase [16], [20] in amyloid plaques. It is interesting to note that even within a single study [38], amyloid plaques have been found either unchanged (after EE only) or decreased (after EE plus cognitive behavioral testing). Different experimental paradigms applied or animal gender may possibly explain such controversial results. The enriched housing paradigm used in the present study, like in Jankowsky et al. [16], [20], consists in a combination of voluntary exercise and cognitive stimulation. In this experimental setting, animals shared access to six exercise wheels in EE, without the formation of hierarchical competition for access to the wheels that might have blunted the benefits of EE. In fact, previous studies indicate that this type of housing is required to achieve beneficial effects on cognition and other outcome measures in AD mice, whereas social and physical activity alone might be insufficient [19]. Female mice housed in EE in the study of Jankowsky et al. [20] showed an increase in plaque formation, while males used by Lazarov et al. [15] displayed a significant reduction. Our experiments have been done on female mice, because enrichment experiments in males evoke territorial behaviour causing stress for subordinate animals and resulting in an increased variability and stress as a confounding variable. We decided to use female mice also because a number of AD studies reported that aging women have a higher risk of developing pathology than men [39], [47] even if in APP^Swe^/PS1^L166P^, females do not exhibit a greater brain amyloid plaque burden than males [30]. Therefore, divergent outcomes observed in male and female could represent a sexually dimorphic response to environmental stimulation. Finally, in the present study and in those of Jankowsky et al. [16], [20], mice were continuously exposed to EE, whereas in Lazarov et al. [15], mice were intermittently exposed to enriched conditions (i.e., several hours daily at first and then 3 h per week). It is thus possible that continuous and chronic exposure to EE, as was presently performed, stimulates the production of Aβ, but that the opposite effect is obtained when the mice are discontinuously exposed to EE. At variance with the majority of other studies, we decided to extend the period of exposure to EE as early in life as possible, including the critical periods of formation and refinement of neural circuits. Although these observations strongly suggest that the metabolism of brain amyloid is affected by EE, further studies are required to unravel the experimental parameters and the mechanisms responsible for this effect.

Notably, transgenic APP^Swe^/PS1^L166P^ mice housed in standard conditions until 2 months of age and then transferred into an “enriched” cage, did not show any difference in Aβ deposition in the intra- or extracellular pool compared to standard housed APP^Swe^/PS1^L166P^ mice. This result indicates that the subsequent rate of Aβ deposition depends on the previous formation, selection and fine tuning of synaptic contacts that took place within the specific time windows of the critical periods. It is interesting to note that even exposure of pregnant mice to enhanced physical activity improved cognitive performance of the offspring [44].

The decrease of plaques following suppression of neuronal electrical activity suggests that the transient enhancement of plaque formation obtained with the EE might be mediated by an increased level of overall neuronal activity in the brain areas involved. This hypothesis is consistent with *in vitro* studies in cultured hippocampal neurons, showing that neuronal stimulation can modulate Aβ homeostasis, enhancing the production of APP cleavage products at the β–secretase site with a consequent increase in Aβ formation [21]–[23]. Actually, this would promote Aβ formation, but also increase production of other fragments such as APP intracellular domain (AICD) which may also modulate synaptic activity, inducing a negative feedback between Aβ deposition and synaptic activity [21]. By chronically suppressing neuronal electrical signalling, we tested the hypothesis that the acceleration in plaque formation obtained by the EE paradigm was mediated by an increase in overall neuronal activity in brain areas involved in cognitive functions. The result of this experiment supports such hypothesis, as chronic infusion of TTX in the hippocampus and associated cortices caused a decrease of the number of amyloid plaques.

Although neuronal activity has been previously proposed to increase Aβ secretion *in vitro* [25], thereby reducing the intracellular deposits, our data do not support this hypothesis. In fact, we did not observe the expected reduction in APP/Aβ intracellular immunoreactivity in APP^Swe^/PS1^L166P^ mice after EE. Consistently, even the chronic suppression of electrical signalling obtained by continuous infusion of TTX did not result in change of APP/Aβ intracellular immunoreactivity. This controversial point might be, at least in part, explained by lack of specificity of most Aβ antibodies including 6E10, the one used in the present study, which does not discriminate between the free/cleaved Aβ peptide versus Aβ epitopes within the APP holoprotein or its other cleaved derivatives [29].

Indeed, APP fragments other than the Aβ peptide, like β C-terminal fragments (β-CTFs), containing the 6E10 epitope, have been shown to be abundant products of a mutated APP transgene, under control of doxycyclin [48]. In this animal model, the APP/Aβ intracellular reactivity of hippocampal pyramidal neurons remained strong for one week following suppression of transcription, while the expression of full length APP and β-CTFs dropped. This suggests that intracellular 6E10 staining is weakly correlated to these derivatives of the APP transgene [48]. However, following 5 weeks of suppression of APP transcription, APP/Aβ intracellular reactivity of hippocampal pyramidal neurons disappeared, while extracellular plaques remained unchanged. Since during suppression of APP transcription the cognitive performance was significantly improved while Aβ peptides and soluble oligomers persisted, Melnikova et al. [48] suggest that other APP fragments like soluble APP ectodomains or CTFs could play a role in cognitive deficits. This conclusion is in line with our findings that cognitive improvements are neither associated with a decrease of plaques nor with a reduction of intracellular APP/Aβ staining.

In conclusion, our results show that a condition, like the EE, that ameliorates the cognitive dysfunction, can have at the same time a worsening effect on amyloid deposition. This confirms that there is a dissociation between the brain load of amyloid plaques and the cognitive deficits [39]. Furthermore, we show that the rate of amyloid plaque formation can be bidirectionally modulated, with an increase in the EE, where mice experience a more intense perceptual, motor and social activity, and a decrease when neuronal activity is suppressed by block of action potentials.

## Supporting Information

Table S1 **Intracellular APP/Aβ load in APP^Swe^/PS1^L166P^ mice is not affected by exposition to EE.**
(DOCX)

Table S2 **Intracellular APP/Aβ load in APP^Swe^/PS1^L166P^ mice is not affected by chronic neuronal activity inhibition by TTX.**
(DOCX)

Text S1 (DOCX)

## References

[1] Rosen WG, Mohs RC, Davis KL (1984) A new rating scale for Alzheimer’s disease Am J Psychiatry 141, 1356–1364. 10.1176/ajp.141.11.1356 6496779

[2] Selkoe DJ (2000) The origins of Alzheimer disease: a is for amyloid JAMA 283, 1615–1617. 10.1001/jama.283.12.1615 10735401

[3] Hardy J (2001) Genetic dissection of primary neurodegenerative diseases Biochem Soc Symp 51–57. 10.1042/bss0670051 11447839

[4] Stern Y, Gurland B, Tatemichi TK, Tang MX, Wilder D, Mayeux R (1994) Influence of education and occupation on the incidence of Alzheimer’s disease JAMA 271, 1004–1010. 8139057

[5] Bonaiuto S, Rocca WA, Lippi A, Giannandrea E, Mele M, Cavarzeran F, Amaducci L (1995) Education and occupation as risk factors for dementia: a population-based case-control study Neuroepidemiology 14, 101–109. 10.1159/000109785 7777124

[6] Friedland RP, Fritsch T, Smyth KA, Koss E, Lerner AJ, Chen CH, Petot GJ, Debanne SM (2001) Patients with Alzheimer’s disease have reduced activities in midlife compared with healthy control-group members Proc Natl Acad Sci U S A 98, 3440–3445. 10.1073/pnas.061002998 PMC30672 11248097

[7] Wilson RS, Bennett DA, Bienias JL, Aggarwal NT, Mendes De Leon CF, Morris MC, Schneider JA, Evans DA (2002) Cognitive activity and incident AD in a population-based sample of older persons Neurology 59, 1910–1914. 10.1212/01.wnl.0000036905.59156.a1 12499482

[8] Colcombe SJ, Kramer AF, McAuley E, Erickson KI, Scalf P (2004) Neurocognitive aging and cardiovascular fitness: recent findings and future directions J Mol Neurosci 24, 9–14. 10.1385/JMN:24:1:009 15314244

[9] Rosenzweig MR, Bennett EL, Krech D (1964) Cerebral Effects of Environmental Complexity and Training among Adult Rats J Comp Physiol Psychol 57, 438–439. 10.1037/h0046387 14155385

[10] Holloway RL Jr (1966) Dendritic branching: some preliminary results of training and complexity in rat visual cortex Brain Res 2, 393–396. 10.1016/0006-8993(66)90009-6 5968207

[11] West RW, Greenough WT (1972) Effect of environmental complexity on cortical synapses of rats: preliminary results Behav Biol 7, 279–284. 10.1016/s0091-6773(72)80207-4 5041867

[12] Greenough WT, Volkmar FR (1973) Pattern of dendritic branching in occipital cortex of rats reared in complex environments Exp Neurol 40, 491–504. 10.1016/0014-4886(73)90090-3 4730268

[13] van Praag H, Kempermann G, Gage FH (2000) Neural consequences of environmental enrichment Nat Rev Neurosci 1, 191–198. 10.1038/35044558 11257907

[14] Arendash GW, Garcia MF, Costa DA, Cracchiolo JR, Wefes IM, et al.. (2004) Environmental enrichment improves cognition in aged Alzheimer’s transgenic mice despite stable β-amyloid deposition Neuroreport 15, 1751–1754. 10.1097/01.wnr.0000137183.68847.4e 15257141

[15] Lazarov O, Robinson J, Tang YP, Hairston IS, Korade-Mirnics Z, et al.. (2005) Environmental enrichment reduces Aβ levels and amyloid deposition in transgenic mice Cell 120, 701–713. 10.1016/j.cell.2005.01.015 15766532

[16] Jankowsky JL, Melnikova T, Fadale DJ, Xu GM, Slunt HH, et al.. (2005) Environmental enrichment mitigates cognitive deficits in a mouse model of Alzheimer’s disease J Neurosci 25, 5217–5224. 10.1523/JNEUROSCI.5080-04.2005 PMC4440804 15917461

[17] Ambree O, Leimer U, Herring A, Gortz N, Sachser N, et al.. (2006) Reduction of amyloid angiopathy and Aβ plaque burden after enriched housing in TgCRND8 mice: involvement of multiple pathways Am J Pathol 169, 544–552. 10.2353/ajpath.2006.051107 PMC1698805 16877355

[18] Berardi N, Braschi C, Capsoni S, Cattaneo A, Maffei L (2007) Environmental enrichment delays the onset of memory deficits and reduces neuropathological hallmarks in a mouse model of Alzheimer-like neurodegeneration J Alzheimers Dis 11, 359–370. 10.3233/jad-2007-11312 17851186

[19] Cracchiolo JR, Mori T, Nazian SJ, Tan J, Potter H, et al.. (2007) Enhanced cognitive activity–over and above social or physical activity–is required to protect Alzheimer’s mice against cognitive impairment, reduce Aβ deposition, and increase synaptic immunoreactivity Neurobiol Learn Mem 88, 277–294. 10.1016/j.nlm.2007.07.007 PMC2083653 17714960

[20] Jankowsky JL, Xu G, Fromholt D, Gonzales V, Borchelt DR (2003) Environmental enrichment exacerbates amyloid plaque formation in a transgenic mouse model of Alzheimer disease J Neuropathol Exp Neurol 62, 1220–1227. 10.1093/jnen/62.12.1220 14692698

[21] Kamenetz F, Tomita T, Hsieh H, Seabrook G, Borchelt D, et al.. (2003) APP processing and synaptic function Neuron. 37, 925–937. 10.1016/s0896-6273(03)00124-7 12670422

[22] Cirrito JR, Yamada KA, Finn MB, Sloviter RS, Bales KR, et al.. (2005) Synaptic activity regulates interstitial fluid amyloid-β levels in vivo Neuron. 48, 913–922. 10.1016/j.neuron.2005.10.028 16364896

[23] Ting JT, Kelley BG, Lambert TJ, Cook DG, Sullivan JM (2007) Amyloid precursor protein overexpression depresses excitatory transmission through both presynaptic and postsynaptic mechanisms Proc Natl Acad Sci U S A 104, 353–358. 10.1073/pnas.0608807104 PMC1765464 17185415

[24] Buckner RL, Snyder AZ, Shannon BJ, LaRossa G, Sachs R, et al.. (2005) Molecular, structural, and functional characterization of Alzheimer’s disease: evidence for a relationship between default activity, amyloid, and memory J Neurosci 25, 7709–7717. 10.1523/JNEUROSCI.2177-05.2005 PMC6725245 16120771

[25] Tampellini D, Rahman N, Gallo EF, Huang Z, Dumont M, et al.. (2009) Synaptic activity reduces intraneuronal Aβ, promotes APP transport to synapses, and protects against Aβ-related synaptic alterations J Neurosci 29, 9704–9713. 10.1523/JNEUROSCI.2292-09.2009 PMC2763626 19657023

[26] Tampellini D, Capetillo-Zarate E, Dumont M, Huang Z, Yu F, et al.. (2010) Effects of synaptic modulation on β-amyloid, synaptophysin, and memory performance in Alzheimer’s disease transgenic mice J Neurosci 30, 14299–14304. 10.1523/JNEUROSCI.3383-10.2010 PMC2972675 20980585

[27] Billings LM, Oddo S, Green KN, McGaugh JL, LaFerla FM (2005) Intraneuronal Aβ causes the onset of early Alzheimer’s disease-related cognitive deficits in transgenic mice Neuron 45, 675–688. 10.1016/j.neuron.2005.01.040 15748844

[28] Knobloch M, Konietzko U, Krebs DC, Nitsch RM (2007) Intracellular Aβ and cognitive deficits precede β-amyloid deposition in transgenic arcAβ mice Neurobiol Aging 28, 1297–1306. 10.1016/j.neurobiolaging.2006.06.019 16876915

[29] Winton MJ, Lee EB, Sun E, Wong MM, Leight S, et al.. (2011) Intraneuronal APP, not free Aβ peptides in 3xTg-AD mice: implications for tau versus Aβ-mediated Alzheimer neurodegeneration J Neurosci 31, 7691–7699. 10.1523/JNEUROSCI.6637-10.2011 PMC3118598 21613482

[30] Radde R, Bolmont T, Kaeser SA, Coomaraswamy J, Lindau D, et al.. (2006) Aβ42-driven cerebral amyloidosis in transgenic mice reveals early and robust pathology EMBO Rep 7, 940–946. 10.1038/sj.embor.7400784 PMC1559665 16906128

[31] Koenig J, Cosquer B, Cassel JC (2008) Activation of septal 5-HT1A receptors alters spatial memory encoding, interferes with consolidation, but does not affect retrieval in rats subjected to a water-maze task Hippocampus 18, 99–118. 10.1002/hipo.20368 17924524

[32] Paxinos G, Franklin KBJ (2001) The mouse brain in stereotaxic coordinates San Diego, CA, USA: Academic Press.

[33] Hyman BT, Van Hoesen GW, Damasio AR (1990) Memory-related neural systems in Alzheimer’s disease: an anatomic study Neurology 40, 1721–1730. 10.1212/wnl.40.11.1721 2234428

[34] Braak H, Braak E, Bohl J, Bratzke H (1998) Evolution of Alzheimer’s disease related cortical lesions J Neural Transm Suppl 54, 97–106. 10.1007/978-3-7091-7508-8_9 9850918

[35] Frisoni GB, Pievani M, Testa C, Sabattoli F, Bresciani L, et al.. (2007) The topography of grey matter involvement in early and late onset Alzheimer’s disease Brain. 130, 720–730. 10.1093/brain/awl377 17293358

[36] Bolmont T, Haiss F, Eicke D, Radde R, Mathis CA, et al.. (2008) Dynamics of the microglial/amyloid interaction indicate a role in plaque maintenance J Neurosci 28, 4283–92. 10.1523/JNEUROSCI.4814-07.2008 PMC3844768 18417708

[37] Bennett JC, McRae PA, Levy LJ, Frick KM (2006) Long-term continuous, but not daily, environmental enrichment reduces spatial memory decline in aged male mice Neurobiol Learn Mem 85, 139–52. 10.1016/j.nlm.2005.09.003 16256380

[38] Costa DA, Cracchiolo JR, Bachstetter AD, Hughes TF, Bales KR, et al.. (2007) Enrichment improves cognition in AD mice by amyloid-related and unrelated mechanisms Neurobiol Aging 28, 831–844. 10.1016/j.neurobiolaging.2006.04.009 16730391

[39] Katzman R, Brown T, Thal LJ, Fuld PA, Aronson M, et al.. (1988) Comparison of rate of annual change of mental status score in four independent studies of patients with Alzheimer’s disease Ann Neurol 24, 384–389. 10.1002/ana.410240306 3228273

[40] Wolfe KJ, Cyr DM (2011) Amyloid in neurodegenerative diseases: friend or foe? Semin Cell Dev Biol 22(5): 476–81. 21458579 10.1016/j.semcdb.2011.03.011 PMC3182296

[41] Adlard PA, Perreau VM, Pop V, Cotman CW (2005) Voluntary exercise decreases amyloid load in a transgenic model of Alzheimer’s disease J Neurosci 25, 4217–4221. 10.1523/JNEUROSCI.0496-05.2005 PMC6725122 15858047

[42] Um HS, Kang EB, Leem YH, Cho IH, Yang CH, et al.. (2008) Exercise training acts as a therapeutic strategy for reduction of the pathogenic phenotypes for Alzheimer’s disease in an NSE/APPsw-transgenic model Int J Mol Med 22, 529–539. 18813861

[43] Wolf SA, Kronenberg G, Lehmann K, Blankenship A, Overall R, et al.. (2006) Cognitive and physical activity differently modulate disease progression in the amyloid precursor protein (APP)-23 model of Alzheimer’s disease Biol Psychiatry 60, 1314–1323. 10.1016/j.biopsych.2006.04.004 16806094

[44] Herring A, Lewejohann L, Panzer AL, Donath A, Kroll O, et al.. (2012) Preventive and therapeutic types of environmental enrichment counteract β amyloid pathology by different molecular mechanisms Neurobiol Dis 42, 530–538. 10.1016/j.nbd.2011.03.007 21406231

[45] Mirochnic S, Wolf S, Staufenbiel M, Kempermann G (2009) Age effects on the regulation of adult hippocampal neurogenesis by physical activity and environmental enrichment in the APP23 mouse model of Alzheimer disease Hippocampus 19, 1008–1018. 10.1002/hipo.20560 19219917

[46] Cotel MC, Jawhar S, Christensen DZ, Bayer TA, Wirths O (2012) Environmental enrichment fails to rescue working memory deficits, neuron loss, and neurogenesis in APP/PS1KI mice Neurobiol Aging 33, 96–107. 10.1016/j.neurobiolaging.2010.02.012 20359774

[47] Jorm AF, Korten AE, Henderson AS (1987) The prevalence of dementia: a quantitative integration of the literature Acta Psychiatr Scand 76, 465–479. 10.1111/j.1600-0447.1987.tb02906.x 3324647

[48] Melnikova T, Fromholt S, Kim HS, Lee D, Xu G, et al.. (2013) Reversible pathologic and cognitive phenotypes in an inducible model of Alzheimer-amyloidosis Neurobiol Dis 33, 3765–3779. 10.1523/JNEUROSCI.4251-12.2013 PMC3711622 23447589

