# Supplementary material for: Early Enriched Environment Exposure Protects Spatial Memory and Accelerates Amyloid Plaque Formation in APPSwe/PS1L166P Mice
Source: PLoS One. 2013 Jul 24;8(7):e69381. doi: 10.1371/journal.pone.0069381 (PMC3722266; doi:10.1371/journal.pone.0069381)
Supplement: Table S1 — Intracellular APP/Aβ load in APPSwe/PS1L166P mice is not affected by exposition to EE. (DOCX) [file pone.0069381.s001.docx]

**Table S1. Intracellular APP/Aβ load in APP^Swe^/PS1^L166P^ mice is not affected by exposition to EE.**

|  |  | **HIP** | | **EC** | | | **PMC** | | |
| --- | --- | --- | --- | --- | --- | --- | --- | --- | --- |
| Age  (months) | Condition | Optical density (AU)  (mean±SEM) | Layer thickness  (µm)  (mean±SEM) | Optical density (AU)  (mean±SEM) | APP/Aβ+cell number  (APP/Aβ+cell number/mm^2^)  (mean±SEM) | APP/Aβ+cell size  (µm^2^)  (mean±SEM) | Optical  density (AU)  (mean±SEM) | APP/Aβ+cell number  (APP/Aβ+cell number/mm^2^)  (mean±SEM) | APP/Aβ+cell  size  (µm^2^)  (mean±SEM) |
| 2 | SE since birth | 37.34 ± 5.55 | 43.65 ± 2.13 | 14.29 ± 1.92 | 141.03 ± 6.89 | 128.79 ± 16.75 | 34.18 ± 1.27 | 245.02 ± 4.86 | 84.44 ± 0.66 |
| 2 | EE since birth | 46.51 ± 5.67 | 44.77 ± 2.40 | 17.43 ± 4.15 | 137.07 ± 4.09 | 98.96 ± 5.09 | 36.28 ± 3.62 | 240.56 ± 10.72 | 97.61 ± 1.83 |
| 4 | SE since birth | 42.16 ± 2.04 | 45.64 ± 1.14 | 20.13 ± 2.15 | 120.97 ± 2.75 | 129.09 ± 2.51 | 46.49 ± 1.72 | 221.12 ± 8.56 | 100.70 ± 2.85 |
| 4 | EE since birth | 50.48 ± 4.09 | 43.80 ± 1.33 | 15.45 ± 3.22 | 128.72 ± 0.67 | 109.72 ± 2.95 | 43.23 ± 1.20 | 244.28 ± 3.47 | 101.83 ± 6.16 |
| 4 | EE since 2 months | 46.15 ± 1.18 | 46.62 ± 2.16 | 20.80 ± 3.85 | 126.64 ± 6.38 | 154.83 ± 11.73 | 38.81 ± 3.86 | 246.90 ± 1.87 | 92.82 ± 4.59 |
| 6 | SE since birth | 24.60 ± 1.17 | 47.83 ± 1.69 | 13.46 ± 2.58 | 103.14 ± 4.85 | 108.20 ± 5.06 | 42.25± 5.62 | 191.22 ± 6.31 | 124.32 ± 4.05 |
| 6 | EE since birth | 20.52 ± 3.43 | 46.49 ± 1.04 | 18.48 ± 1.01 | 104.03 ± 3.94 | 94.56 ± 11.28 | 30.40 ± 1.24 | 200.45 ± 12.22 | 128.68 ± 5.44 |

(n=5 in each group, One-way ANOVA, *P*˃0.05)

(HIP: hippocampus; EC: Entorhinal Cortex; PMC: Primary Motor Cortex; SE:Standard Environment; EE: Enriched Environment)
