# Supplementary material for: Early Enriched Environment Exposure Protects Spatial Memory and Accelerates Amyloid Plaque Formation in APPSwe/PS1L166P Mice
Source: PLoS One. 2013 Jul 24;8(7):e69381. doi: 10.1371/journal.pone.0069381 (PMC3722266; doi:10.1371/journal.pone.0069381)
Supplement: Table S2 — Intracellular APP/Aβ load in APPSwe/PS1L166P mice is not affected by chronic neuronal activity inhibition by TTX. (DOCX) [file pone.0069381.s002.docx]

**Table S2. Intracellular APP/Aβ load in APP^Swe^/PS1^L166P^ mice is not affected by chronic neuronal activity inhibition by TTX.**

|  | **HIP** | | **EC** | | |
| --- | --- | --- | --- | --- | --- |
| Condition | Optical density (AU)  (mean±SEM) | Layer thickness  (µm)  (mean±SEM) | Optical density  (AU)  (mean±SEM) | APP/Aβ+cell number  (APP/Aβ+cell number/mm^2^)  (mean±SEM) | APP/Aβ+cell size  (µm^2^)  (mean±SEM) |
| VEHICLE | 1.07 ± 0.08 | 1.03 ± 0.04 | 1.07 ± 0.35 | 1.00 ± 0.06 | 0.97 ± 0.04 |
| TTX | 0.98 ± 0.04 | 1.04 ± 0.04 | 1.28 ± 0.48 | 1.06 ± 0.04 | 0.98 ± 0.03 |

(n=5 in each group, One-way ANOVA, *P*˃0.05)

(HIP: hippocampus; EC: Entorhinal Cortex; TTX: Tetrodotoxin; SE:Standard Environment; EE: Enriched Environment)
