# Supplementary material for: Early Enriched Environment Exposure Protects Spatial Memory and Accelerates Amyloid Plaque Formation in APPSwe/PS1L166P Mice
Source: PLoS One. 2013 Jul 24;8(7):e69381. doi: 10.1371/journal.pone.0069381 (PMC3722266; doi:10.1371/journal.pone.0069381)
Supplement: Text S1 — (DOCX) [file pone.0069381.s003.docx]

*1 Behavioural tests*

*1.1 Morris water maze test*

The maze consisted of a circular pool (140 cm diameter; 40 cm height) filled with water at 25° C to avoid hypothermia added with powder of milk to hide a submerged platform. A small escape platform made of transparent plastic (15 cm diameter) was placed in the center of one quadrant at a fixed position and was hidden 1 cm beneath the water surface. The test consisted of two steps: the acquisition phase (days 1-4) and the probe trial (day 5). The acquisition phase consisted of 4 training days (days 1-4) with four trials per day with a 15 minutes inter-trial interval. Four points equally spaced along the circumference of the pool (north, south, west, east) served as the starting position, which was randomized across the four trials each day. For each trial, the mouse was released from the side of the pool, facing the wall. If an animal did not reach the platform within 90 seconds, it was guided to the platform, where it was allowed to rest for 30 seconds, before being returned to its home cage. Mice were kept dry, between trials, in a cage filled with paper towels. The escape latencies were recorded. On day 5, a probe trial was performed to assess spatial memory after a 24 hours delay since the last acquisition trial. In the probe trial, the platform was removed from the maze and the animals were allowed to swim freely for 60 seconds. The time spent in each quadrant (quadrant 1, Q_1_; quadrant 2, Q_2_; quadrant 3, Q_3_ and target quadrant, Q_T_, in which the escape platform was located during the acquisition phase) was recorded. The primary retention measure was the accuracy ratio (AR) (31) calculated as follows: time spent in the target quadrant (T_QT_) multiplied by 3 and divided by the time spent in the other three quadrants (T_Q1_ + T_Q2_ + T_Q3_). An AR of 1 corresponds to chance level, indicating no preference for Q_T_ relative to the other quadrants. An AR of 2 corresponds to 24 s spent in Q_T_, while an accuracy ratio of 3 corresponds to 30 s in Q_T_. In the probe trial, also swim velocity and distance were assessed. Data were analyzed in the digitized image using a computerized video-tracking software (Ethovision XT video track system; Noldus Information Technology, Wageningen, The Netherlands).

*1.2 Eight arm radial maze test*

The maze consisted of eight identical arms extending radially from an octagonal central area. At the end of each arm the mice received a sweetened water reward (saccharine solution). Before testing, the mice were deprived of water for 24 h and then their access to water was restricted to 2 h per day for the duration of testing. For each session, all arms were baited with saccharine solution, and animals were permitted to enter in all arms from the octagonal central area until the eight rewards had been consumed. Water-restricted APP^Swe^/PS1^L166P^ mice and their wild-type littermates were given one training session per day for ten consecutive days and after 3 days of adaptation, the number of errors (entries into previously visited arms) in each session was recorded for seven consecutive days.
